# Trend analysis of major cancer statistics according to sex and severity levels in Korea

**DOI:** 10.1371/journal.pone.0203110

**Published:** 2018-09-13

**Authors:** Minsu Ock, Woong Jae Choi, Min-Woo Jo

**Affiliations:** 1 Department of Preventive Medicine, Ulsan University Hospital, University of Ulsan College of Medicine, Ulsan, South Korea; 2 School of Biosystem and Biomedical Science, Korea University, Seoul, South Korea; 3 Department of Preventive Medicine, University of Ulsan College of Medicine, Seoul, South Korea; Universidad Miguel Hernandez de Elche, SPAIN

## Abstract

Existing epidemiologic reports or studies of cancer statistics in Korea lack sufficient data on cancer severity distributions and observed survival rates. This study analyzed trends in major cancer statistics according to sex and severity levels in Korea from 2006 to 2013. We included eight cancers (hepatocellular carcinoma, and thyroid, colorectal, gastric, lung, prostate, breast, and cervical cancer), using Korea Central Cancer Registry data. Severity level was classified by Surveillance, Epidemiology, and End Results (SEER) stage as follows: localized, regional, distant, or unknown. Numbers of incident cancer cases from 2006 to 2013 were described by sex and SEER stage. We estimated up to 8-year observed survival rates of major cancers by sex and SEER stage, and provided prevalence rates by sex and SEER stage in 2011, 2012, and 2013. Although increases in new cancer cases are slowing and the total number of incident cancer cases in 2013 decreased for the first time since 2006, the number of prevalent cancer cases was 663,530 in 2013, an increase of 13.3% compared to 2011. Among the five cancers affecting both sexes, sex-related differences in 5-year observed survival rates for lung cancer were greatest in the localized stage (men, 31.9%; women, 48.1%), regional stage (men, 20.0%; women, 31.3%), and unknown stage (men, 24.3%; women, 37.5%). The sum of the proportions of localized and regional stages for thyroid and breast cancer was over 90% in 2013, while the sum of the proportions of localized and regional stages for lung cancer was only 56.7% in 2013. Differences in observed survival rates between men and women were prominent in lung cancer for all SEER stages. The reported epidemiologic data from this study can be used to obtain a more valid measure of cancer burden using a summary measure of population health.

## Introduction

The burden of cancer is substantial not only in developed countries but also in developing countries [1]. In 2013, 8.2 million people died from cancer, 14.9 million people were newly diagnosed with cancer, and cancer was the cause of 196.3 million disability-adjusted life years (DALYs) worldwide [2]. The incidence of cancer is expected to increase continuously and strain the world’s healthcare resources owing to population growth and aging [3]. In South Korea (hereinafter Korea), cancer is the leading cause of death. In 2013, there were 75,334 deaths due to cancer, and 225,343 cancer cases were newly diagnosed [4]. Furthermore, the burden of cancer in 2012 was 3,471.79 DALYs per 100,000 persons [5] and DALYs due to all neoplasms accounted for 8.44% of total DALYs [6].

Measuring the disease burden is essential for proper allocation of healthcare resources [7]. In the case of cancer, it is also important to measure the cancer burden to determine priorities for healthcare services and research. In this context, generating accurate cancer statistics is required to establish cancer control and prevention strategies [4]. Accordingly, many countries such as the United States [8], Japan [9], Canada [10], and Australia [11] have been attempting to improve the collection and analysis of cancer data and release annual reports of national cancer statistics. Korea also publishes annual reports of cancer statistics, including incidence rates, mortality rates, relative survival rates, and prevalence rates by sex [4], and several studies have provided descriptive epidemiology of various cancers [12–15].

However, existing epidemiologic reports or studies of cancer statistics in Korea have two limitations. First, data regarding cancer severity distributions were insufficient, and only incidence rates of cancer based on the Surveillance, Epidemiology, and End Results (SEER) stage were available. When estimating DALYs due to cancer using the prevalence-based approach, the main method of the study of global burden of disease, prevalence data regarding severity distribution are required to accurately calculate the DALYs [16]. Second, survival data in annual reports and previous studies were based on relative survival rates, not observed survival rates. In terms of relative survival rates, values above 100% can be estimated in some groups of patients, and this result appears counterintuitive for patients. Furthermore, observed survival rates by severity level provide patients with more accurate prognostic information for cancer, compared to overall relative survival rates.

In the present study, we analyzed the trends in major cancer statistics in Korea according to sex and severity levels from 2006 to 2013. Specifically, we described the number of incident cancer cases by sex and SEER stage from 2006 to 2013. Furthermore, we estimated up to 8-year observed survival rates of major cancers by sex and SEER stage. We also provided the 5-year prevalence rates of major cancers by sex and SEER stage in 2011, 2012, and 2013.

## Materials and methods

A total of eight cancers (hepatocellular carcinoma, thyroid cancer, colorectal cancer, gastric cancer, lung cancer, prostate cancer, breast cancer, and cervical cancer) were included in the present study.

### Data

We used data from the Korea Central Cancer Registry (KCCR) of the National Cancer Center Korea. More than 190 hospitals participate in the KCCR, and data regarding over 90% of newly diagnosed cancers in Korea are collected [17]. The KCCR offers annual national cancer incidence, survival, and prevalence data, and the KCCR database includes information regarding patients with cancer, such as sex, age, and date of diagnosis [4]. From the KCCR, we obtained the number of cancer patients by sex, age, and severity level from 2006 to 2013, as well as follow-up data for mortality for up to eight years. Furthermore, we utilized mid-year population data based on resident registration from the Korean Statistical Information Service of Statistics Korea to calculate incidence rates and prevalence rates [18]. The severity level was classified by SEER stage as follows: localized stage, regional stage, distant stage, and unknown stage. The SEER stage was based on the time of diagnosis.

### Analysis

First, we described the number of incident cancer cases according to sex and SEER stage from 2006 to 2013. The incidence rates by sex and SEER stage were determined as the number of incident cancer cases by sex and SEER stage divided by the mid-year population by sex. The means of incidence rates and their 95% confidence intervals from 2006 to 2013 according to Poisson distribution assumption were estimated by type of cancer, sex, and SEER stage. Furthermore, we conducted statistical tests for linear trend of overall incidence rates and proportions of incidence rates by stage.

Patients with cancer identified in 2006 underwent follow-up for all-cause mortality and observed survival rates by sex and SEER stage. All-cause mortality and observed survival rates by sex and SEER stage in each follow-up year were calculated as the number of patients with cancer alive by sex and SEER stage in each follow-up year divided by the total number of patients with cancer by sex and SEER stage in 2006.

**Fig 1:**
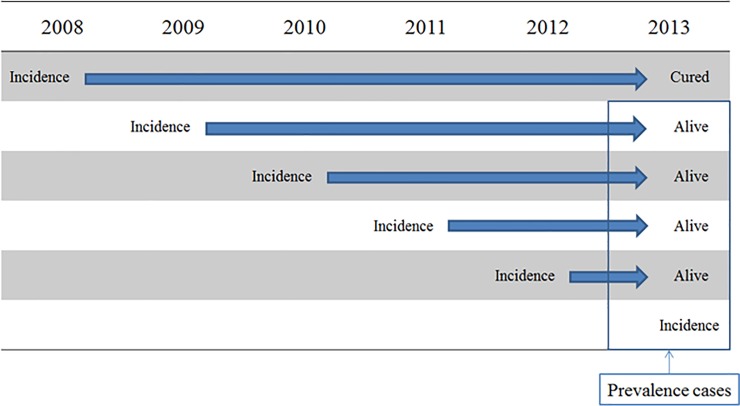
Method of estimating prevalent cases.

Finally, we estimated the prevalence rates for eight cancers by sex and SEER stage in 2011, 2012, and 2013. Fig 1 shows the method of estimating the number of prevalent cases. We assumed that patients with cancer who lived more than five years past their diagnosis were recovered from the cancer, and these patients were excluded from the prevalent cases. For example, prevalent cases in 2013 included the patients with cancer who were diagnosed in 2013, as well as patients with cancer who were diagnosed since 2009 and still alive (Fig 1). As with incidence rates, the 5-year prevalence rates by sex and SEER stage were calculated as the number of prevalent cases by sex and SEER stage divided by the mid-year population by sex.

In the case of breast cancer, we only analyzed the female patients, because male patients account for a small proportion of the total patients with breast cancer. For a similar reason, all analyses were restricted to individuals aged ≥ 30 years. In particular, in the case of prostate cancer, only individuals aged ≥ 50 were included in the analyses, because prostate cancer patients under age 50 are rare in Korea.

We used Microsoft Office Excel 2010 and Stata software (Stata/SE 13.1) for all analyses. In this study, *P*-values less than 0.05 were regarded statistically significant.

### Ethical approval

Ethical approval and consent to participate were unnecessary because we used publicly available data without any personal identifiers.

## Results

### Incident cancer cases

**Fig 2:**
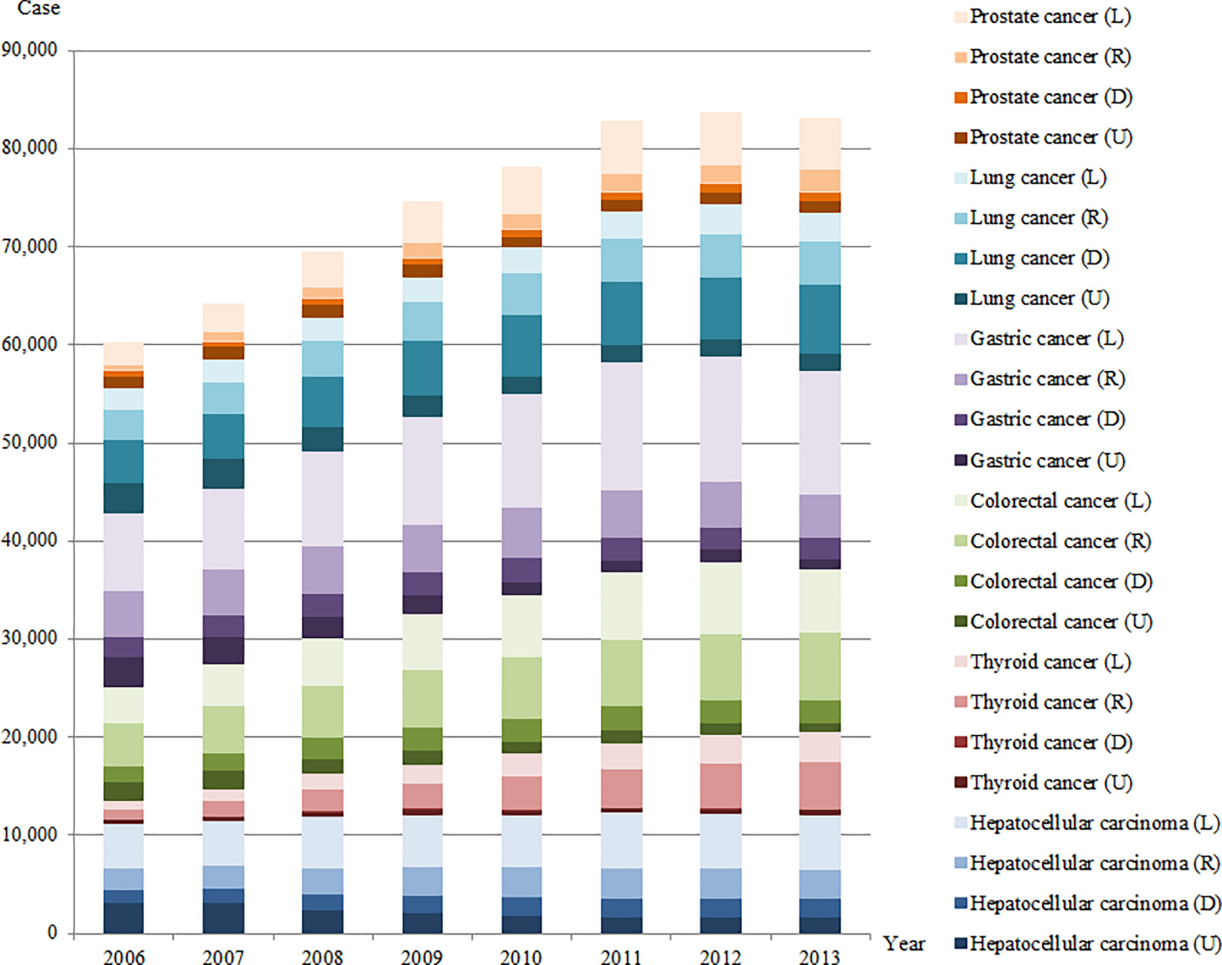
Number of incident cases for six cancers in men according to SEER stage from 2006 to 2013.

**Fig 3:**
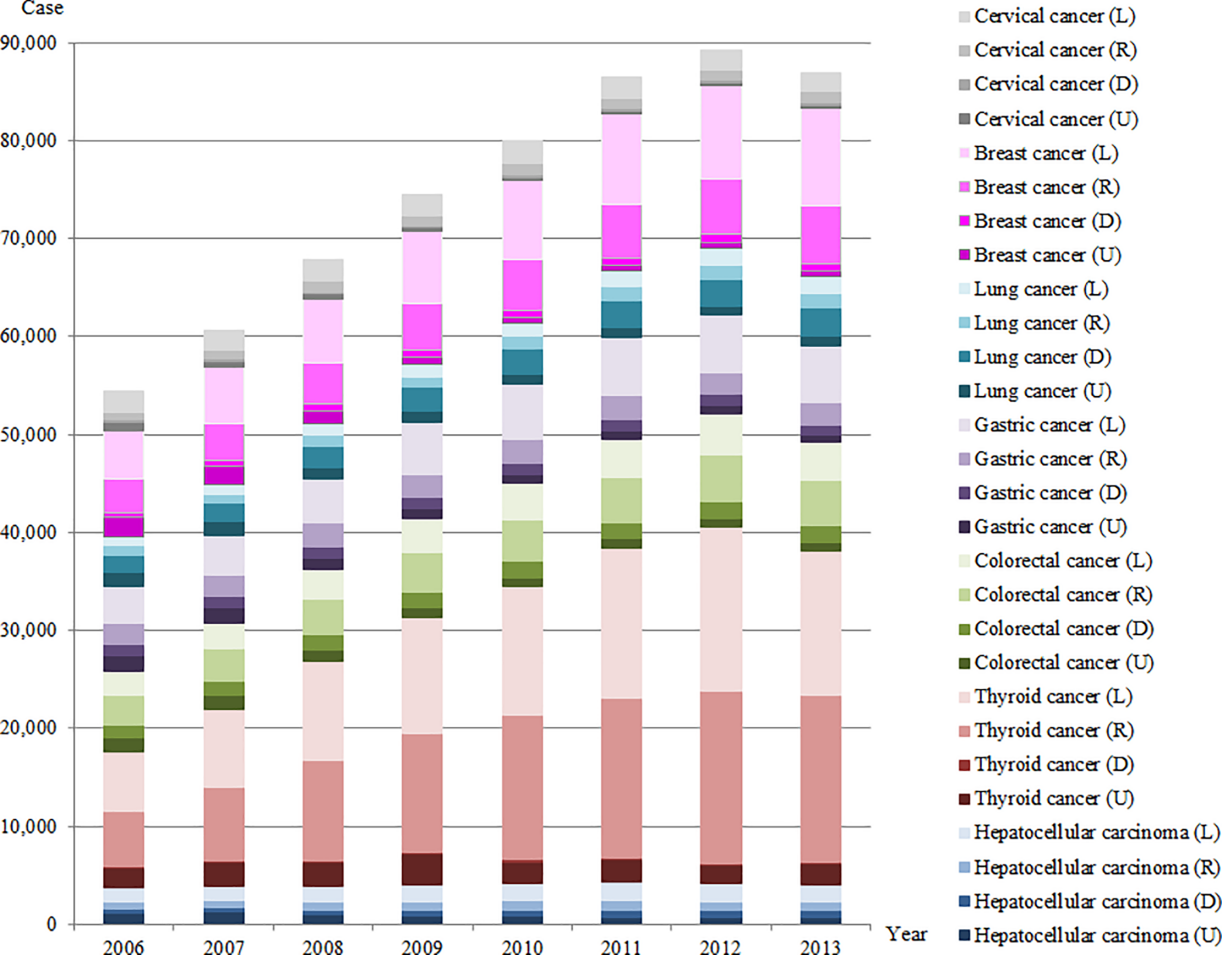
Number of incident cases for seven cancers in women according to SEER stage from 2006 to 2013.

Figs 2 and 3 show the proportions of incident cancer cases for a total of eight cancers by sex and SEER stage from 2006 to 2013. S1 File shows additional details of the number of incident cancer cases, mean incidence rates, and statistical tests for linear trend of overall incidence rates and proportions of incidence rates by SEER stage (S1 File). The total number of newly diagnosed cancer cases between 2006 and 2013 increased by 22,966 and 32,525 cases for men (from 60,138 to 83,104) and women (from 54,462 to 86,987), respectively. However, increases in incident cancer cases are slowing, and the number of newly diagnosed cancer cases in 2013 decreased for the first time since 2006.

In men, the most common cancer in 2013 was gastric cancer (20,266), followed by colorectal cancer (16,593), and lung cancer (16,171). The incidence rate per 100,000 population was higher in the order of gastric cancer (125.7), prostate cancer (125.4), and colorectal cancer (102.9). The proportion of local stage in 2013 was largest in gastric cancer (62.3%), followed by prostate cancer (55.0%) and hepatocellular carcinoma (45.7%). The proportion of distant stage in 2013 was largest in lung cancer (43.7%), followed by hepatocellular carcinoma (15.0%) and colorectal cancer (14.2%). In gastric cancer, the proportion of local stage increased by 17.5 percentage points between 2006 and 2013 (*P*-trend <0.001). However, the proportion of distant stage in the same period increased by 9.9 percentage points in lung cancer (*P*-trend <0.001).

In women, the most common cancer in 2013 was thyroid cancer (34,087), followed by breast cancer (17,231) and colorectal cancer (11,025). The incidence rate per 100,000 population was higher in the order of thyroid cancer (201.9), breast cancer (102.0), and colorectal cancer (65.3). The proportion of local stage in 2013 was largest in gastric cancer (58.8%), followed by breast cancer (58.0%) and cervical cancer (54.5%). However, the proportion of distant stage in 2013 was largest in lung cancer (41.7%), followed by hepatocellular carcinoma (15.9%) and colorectal cancer (15.8%). Although the proportion of the local stage in lung cancer between 2006 and 2013 increased by 6.7 percentage points (*P*-trend <0.001), the proportion of the distant stage in lung cancer during the same period increased by 5.6 percentage points (*P*-trend <0.001).

### Survival rates

**Fig 4:**
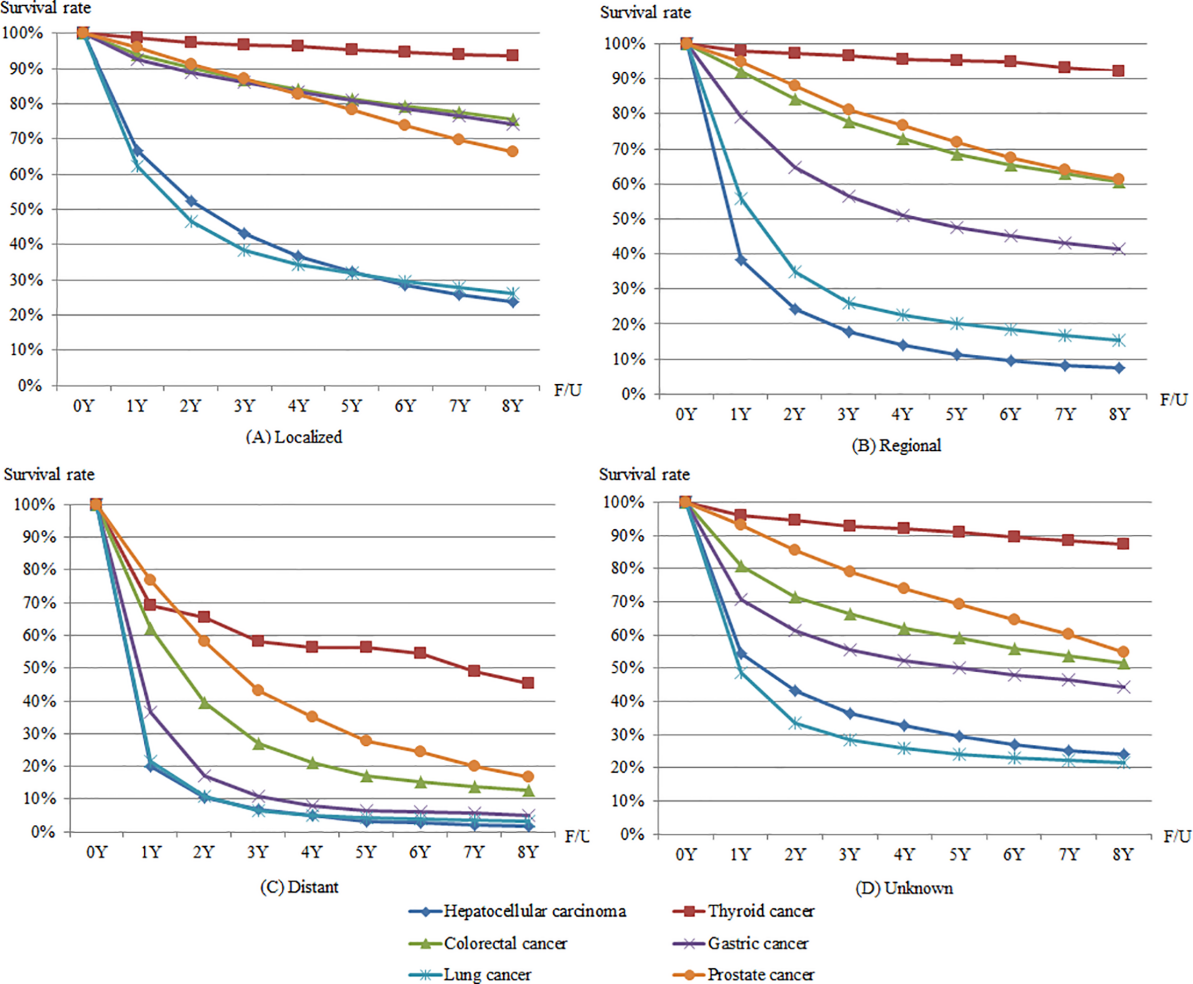
8-year observed survival rates for six cancers in men by SEER stage.

Fig 4 shows 8-year observed survival rates for men by SEER stage. Among the six cancers, the 5-year observed survival rate of thyroid cancer was highest in all SEER stages: 95.3% (localized stage), 95.2% (regional stage), 56.4% (distant stage), and 90.9% (unknown stage). The 5-year observed survival rate for lung cancer was lowest in localized stage (31.9%), while 5-year observed survival rates for colorectal cancer and gastric cancer were over 80% in localized stage (81.5% and 81.1%, respectively). In regional stage and distant stage, the 5-year observed survival rate was lowest for hepatocellular carcinoma (11.3% and 3.2%, respectively).

**Fig 5:**
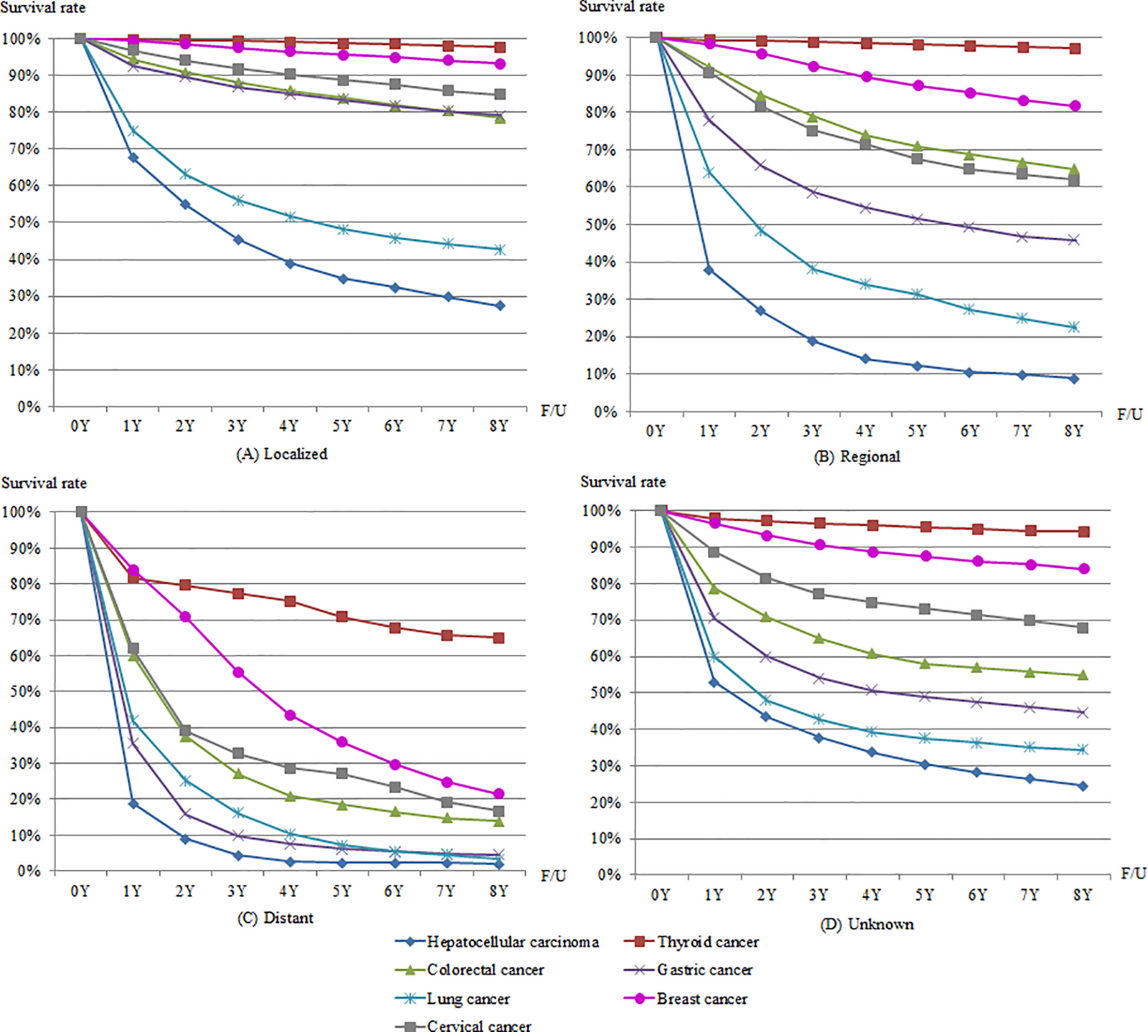
8-year observed survival rates for seven cancers in women by SEER stage.

Fig 5 shows 8-year observed survival rates for women by SEER stage. Among the seven cancers, the 5-year observed survival rate of thyroid cancer was highest in all SEER stages: 98.7% (localized stage), 98.1% (regional stage), 70.8% (distant stage), and 95.5% (unknown stage). The 5-year observed survival rate of hepatocellular carcinoma was lowest in all SEER stages: 34.8% (localized stage), 12.2% (regional stage), 2.3% (distant stage), and 30.4% (unknown stage). Among the five cancers affecting both men and women, the differences in the 5-year observed survival rates of lung cancer were greatest in localized stage (31.9% in men and 48.1% in women), regional stage (20.0% in men and 31.3% in women), and unknown stage (24.3% in men and 37.5% in women). In the case of distant stage, the differences in the 5-year observed survival rates of thyroid cancer were greatest (56.4% in men and 70.8% in women). S2 File shows additional details for observed survival rates of up to eight years by sex and SEER stage (S2 File).

### 5-year prevalence rates

Table 1 shows the trends in overall 5-year prevalence rates of cancers by sex and SEER stage from 2011 to 2013. The number of prevalent cancer cases was 663,530 in 2013, which represented a 13.3% increase compared to 2011. The sums of proportions of localized stage and regional stage for thyroid cancer and breast cancer were over 90% in 2013, while the sum of proportions of localized stage and regional stage for lung cancer was only 56.7% in 2013.

**Table 1:** Number of cancer cases and prevalence rates of cancers by sex and SEER stage from 2011 to 2013.

Cancer	Stage	2011				2012				2013			
		Men		Women		Men		Women		Men		Women	
		N	Rate^a^	N	Rate^a^	N	Rate^a^	N	Rate^a^	N	Rate^a^	N	Rate^a^
Hepatocellular carcinoma	Localized	17,633	113.2	5,859	35.9	18,731	118.1	6,085	36.6	19,502	120.9	6,277	37.2
	Regional	6,233	40.0	1,901	11.6	6,548	41.3	1,952	11.7	6,754	41.9	2,071	12.3
	Distant	2,615	16.8	930	5.7	2,605	16.4	969	5.8	2,484	15.4	930	5.5
	Unknown	5,668	36.4	2,437	14.9	5,117	32.2	2,226	13.4	4,858	30.1	2,139	12.7
	Total	32,149	206.4	11,127	68.2	33,001	208.0	11,232	67.6	33,598	208.3	11,417	67.6
Thyroid cancer	Localized	9,502	61.0	58,221	356.7	11,324	71.4	66,821	402.1	12,744	79.0	71,361	422.6
	Regional	13,489	86.6	60,721	372.0	16,499	104.0	70,773	425.9	19,105	118.5	77,613	459.6
	Distant	295	1.9	855	5.2	299	1.9	878	5.3	292	1.8	852	5.0
	Unknown	2,407	15.4	11,846	72.6	2,487	15.7	11,333	68.2	2,511	15.6	11,074	65.6
	Total	25,693	164.9	131,643	806.5	30,609	192.9	149,805	901.5	34,652	214.9	160,900	952.8
Colorectal cancer	Localized	26,136	167.8	15,416	94.4	29,057	183.1	16,987	102.2	30,414	188.6	17,812	105.5
	Regional	25,434	163.3	17,182	105.3	27,137	171.0	18,453	111.0	28,522	176.8	19,268	114.1
	Distant	5,689	36.5	3,924	24.0	5,902	37.2	4,083	24.6	5,907	36.6	4,120	24.4
	Unknown	5,622	36.1	3,981	24.4	4,947	31.2	3,559	21.4	4,413	27.4	3,245	19.2
	Total	62,881	403.6	40,503	248.1	67,043	422.5	43,082	259.3	69,256	429.4	44,445	263.2
Gastric cancer	Localized	49,667	318.8	23,671	145.0	54,142	341.2	25,638	154.3	57,037	353.6	26,904	159.3
	Regional	17,676	113.5	8,722	53.4	17,676	111.4	8,685	52.3	17,468	108.3	8,710	51.6
	Distant	3,981	25.6	1,924	11.8	3,973	25.0	1,921	11.6	3,737	23.2	1,783	10.6
	Unknown	5,940	38.1	3,570	21.9	4,948	31.2	2,983	18.0	4,196	26.0	2,696	16.0
	Total	77,264	495.9	37,887	232.1	80,739	508.8	39,227	236.1	82,438	511.1	40,093	237.4
Lung cancer	Localized	8,043	51.6	4,709	28.9	8,889	56.0	5,477	33.0	9,528	59.1	6,185	36.6
	Regional	10,291	66.1	3,842	23.5	10,921	68.8	4,230	25.5	11,434	70.9	4,552	27.0
	Distant	9,517	61.1	5,112	31.3	9,844	62.0	5,312	32.0	10,640	66.0	5,673	33.6
	Unknown	5,329	34.2	3,516	21.5	4,876	30.7	3,242	19.5	4,711	29.2	3,153	18.7
	Total	33,180	213.0	17,179	105.2	34,530	217.6	18,261	109.9	36,313	225.1	19,563	115.8
Breast cancer	Localized	-	-	36,232	222.0	-	-	40,053	241.0	-	-	43,594	258.2
	Regional	-	-	22,570	138.3	-	-	24,260	146.0	-	-	25,807	152.8
	Distant	-	-	2,369	14.5	-	-	2,547	15.3	-	-	2,621	15.5
	Unknown	-	-	4,900	30.0	-	-	3,715	22.4	-	-	2,955	17.5
	Total	-	-	66,071	404.8	-	-	70,575	424.7	-	-	74,977	444.0
Cervical cancer	Localized	-	-	10,264	62.9	-	-	10,303	62.0	-	-	10,135	60.0
	Regional	-	-	4,198	25.7	-	-	4,270	25.7	-	-	4,323	25.6
	Distant	-	-	764	4.7	-	-	784	4.7	-	-	826	4.9
	Unknown	-	-	1,746	10.7	-	-	1,462	8.8	-	-	1,205	7.1
	Total	-	-	16,972	104.0	-	-	16,819	101.2	-	-	16,489	97.6
Prostate cancer	Localized	19,149	275.5	-	-	21,505	295.2	-	-	23,100	304.3	-	-
	Regional	6,817	98.1	-	-	7,779	106.8	-	-	8,733	115.1	-	-
	Distant	2,213	31.8	-	-	2,408	33.0	-	-	2,567	33.8	-	-
	Unknown	5,035	72.4	-	-	4,962	68.1	-	-	4,989	65.7	-	-
	Total	33,214	477.8	-	-	36,654	503.1	-	-	39,389	519.0	-	-

^a^Per 100,000 population

In 2011, the highest prevalence rate per 100,000 population in men was for gastric cancer (495.9), followed by prostate cancer (477.8) and colorectal cancer (403.6). In 2013, the highest prevalence rate per 100,000 population in men was for prostate cancer (519.0), followed by gastric cancer (511.1) and colorectal cancer (429.4). In 2011, the highest prevalence rate per 100,000 population in women was for thyroid cancer (806.5), followed by breast cancer (404.8) and colorectal cancer (248.1). In 2013, the highest prevalence rate per 100,000 population in women was for thyroid cancer (952.8), followed by breast cancer (444.0) and colorectal cancer (263.2). Among five cancers affecting both men and women, thyroid cancer was about 4.4 times more common in women, while hepatocellular carcinoma, colorectal cancer, gastric cancer, and lung cancer were more common in men.

## Discussion

In the present study, we analyzed the number of incident cancer cases, observed survival rates, and prevalence rates by sex and SEER stage for eight cancers (hepatocellular carcinoma, thyroid cancer, colorectal cancer, gastric cancer, lung cancer, prostate cancer, breast cancer, and cervical cancer) from 2006 to 2013 in Korea. The main strength of this study is that we described national cancer statistics for major cancers considering the severity level of the cancer. Because data regarding severity level are often scarce [16, 19], the results from this study will be helpful to allow more accurate estimation of the scale of the burden of disease. When collecting national epidemiologic data for other diseases, such as diabetes mellitus and asthma, it will be necessary to collect information regarding severity level using a functional scale.

Another strength of the present study is that we provided up to 8-year observed survival rates by gender and SEER stage. Following a cancer diagnosis, patient often wonder about their prognosis, including successful treatment rates and survival rates [20]. Accurate information concerning disease prognosis is necessary to enable cancer patients to select treatment options and plan their own lives. The results from this study regarding observed survival rates will assist medical professionals in providing more accurate prognostic information about their disease to patients with cancer, compared to overall relative survival rates. Most of the previous studies regarding cancer statistics in Korea have reported relative survival rates, not observed survival rates [4, 12–15]. Although a relative survival rate has the advantage of evaluating the results of cancer treatment, it also leads cancer patients to overestimate their survival rates. Furthermore, relative survival rates above 100% can be calculated in a cancer with a favorable prognosis, such as thyroid cancer [21], and such values can cause difficulty for cancer patients in understanding their prognosis.

When comparing cancers in terms of proportion of SEER stage in the incident cancer cases, the proportions of distant stage were larger in lung cancer, hepatocellular carcinoma, and colorectal cancer than in other cancers. Efforts should be made to detect these cancers at an early stage. In particular, the proportions of distant stage for lung cancer in 2013 were 43.7% in men and 41.7% in women; these proportions have increased compared to 2006 (*P*-trend <0.001). Based on these results, we may assume that a fair number of patients with lung cancer had inoperable disease at the time of diagnosis. It is known that lung cancer screening in high-risk groups can reduce lung cancer mortality, although several issues, including radiation risk, overdiagnosis bias, and validity of screening method, might be reviewed [22]. If lung cancer screening guidelines are adopted in Korea, distributions of severity level in lung cancer can be monitored to evaluate the effectiveness of those screening guidelines.

On the other hand, in the case of gastric cancer, the proportion of local stage tended to increase and the proportion of distant stage tended to decrease. The proportions of localized stage for gastric cancer in 2006 were 44.8% in men and 43.3% in women, whereas theses proportions in 2013 were 62.3% in men and 58.8% in women (*P*-trend <0.001). However, the proportions of distant stage for gastric cancer in 2006 were 12.1% in men and 12.3 in women, whereas theses proportions in 2013 were 10.4% in men and 10.6% in women (*P*-trend <0.001). One of the hypotheses that can explain these changes is that the Korean National Cancer Screening Program for gastric cancer is effective. It is also reported that the Korean National Cancer Screening Program for gastric cancer has reduced the gastric cancer mortality [23]. However, since these changes are not prominent in other cancers, there may be limitations in estimating the effect of the overall Korean National Cancer Screening Program.

Another noticeable finding is the rapid statistically significant increase in the both incidence of thyroid cancer cases and incidence rates of thyroid cancer. However, the majority of thyroid cancers were either local stage or regional stage in both men and women. The proportion of distant stage for thyroid cancer in 2013 was only 0.8% in men and 0.5% in women, respectively. Furthermore, there were no differences in observed survival rates between localized stage and regional stage. Accordingly, prevalence rates of thyroid cancer have also been increasing recently in both men and women, as determined by the present study. Consistent with the findings of previous studies [21, 24], these results can be explained as overdetection or overdiagnosis of thyroid cancer in Korea. A national effort to reduce unnecessary use of ultrasonography screening in the asymptomatic general population is required.

In general, the observed survival rates in men were lower than those in women. The differences in observed survival rates between men and women were particularly prominent in lung cancer for all SEER stages. The 5-year observed survival rate of lung cancer in the localized stage was 31.9% in men and 48.1% in women, respectively. Differences in the histological type of lung cancer are considered as a plausible explanation for these differences [25], but there has also been speculation that men with lung cancer might be more likely to be current or former smokers than women with lung cancer, which could contribute to the presence of more comorbid conditions in male patients than in female patients [26]. In Korea, the current smoking rate in men (42.1%) was seven times higher than that in women (6.2%) in 2013 [27]. Although the current smoking rate in men has decreased since 2011, a more aggressive smoking policy to prohibit smoking in men is required to reduce the gap in these lung cancer survival rates, and gender-sensitive tobacco control policies are needed [28].

Although increases in new cancer cases are slowing and the total number of incident cancer cases in 2013 decreased for the first time since 2006, the number of prevalent cancer cases was 663,530 in 2013, an increase of 13.3% compared to 2011. In most cancers, the prevalence rates showed steady increases between 2011 and 2013. The trends were the same when thyroid cancer was excluded from the prevalent cancer cases. The increased total prevalence rates of cancers might most likely be due to increased prevalence rates in localized and regional stages. For example, the prevalence rate of breast cancer in the localized stage increased from 222.0 (per 100,000) to 258.2 between 2011 and 2013, but the prevalence rate of breast cancer in the distant stage increased from 14.5 to 15.5 during the same period. The prevalence rate is expected to rise continuously, considering the increasing availability of more effective treatment and diagnosis. Accordingly, evaluation and improvement of health-related quality of life in patients with cancer will become another major issue in Korea [29–31].

This study had several limitations. First, not all cancers were included in this study. We only focused on eight major cancers, which were known for their high incidence, and other cancers including leukemia, kidney cancer, and pancreatic cancer were omitted from the present study. Further study on these cancers is required. Second, analyses in this study were restricted to individuals aged ≥ 50 years for prostate cancer and individuals aged ≥ 30 for other cancers. However, this restriction would not be significant considering that it affects a small proportion of the total number of patients with cancer. Third, we estimated the 5-year prevalence of cancers owing to data limitations and general perceptions of cancer survival. This could mean that cancer survivors’ mortality might not be affected by their cancer after five years’ survival. Further studies will be needed to explore the mortality of cancer survivors beyond five years, as well as long-term follow-up data on cancer survival by SEER stage. Fourth, the cause of death could not be identified in this study. Therefore, only the all-cause mortality rate was reported, and the cause-specific mortality rate could not be reported. Fifth, this study focused on descriptive analyses. Further studies will be required to determine the factors that influence the increase or decrease of incidence and prevalence rates and the changes in severity distribution by sex and type of cancer. Sixth, we did not consider changes in population structure in this study. The reason for this is that the overall numbers of incidence and prevalence cases reported in this study are meaningful in calculating the burden of cancer. In future studies, it would be meaningful to calculate the age standardized incidence and prevalence rates according to changes in population structure in Korea.

## Conclusions

In this study, we analyzed the trends in major cancer statistics according to SEER stage from 2006 to 2013 in Korea. Although increases in new cancer cases are slowing and the total number of incident cancer cases in 2013 decreased for the first time since 2006, the number of prevalent cancer cases was 663,530 in 2013, an increase of 13.3% compared to 2011. In most cancers, the prevalence rates showed steady increases between 2011 and 2013. When comparing cancers in terms of proportion of SEER stage in the incident cancer cases, the proportions of distant stage were larger in lung cancer, hepatocellular carcinoma, and colorectal cancer than in other cancers. The differences in observed survival rates between men and women were particularly prominent in lung cancer for all SEER stages. The reported prevalence rates from this study can be used to obtain a more valid measure of cancer burden using a summary measure of population health, such as DALY and quality-adjusted life year. Furthermore, it will be possible to perform additional studies estimating cancer-specific quality-adjusted life expectancy using the data regarding observed survival rates from this study.

## Supporting information

S1 File Number of incident cancer cases and mean incidence rates by sex. (DOCX)

S2 File Absolute survival rates of up to eight years by sex. (DOCX)
