# Supplementary material for: Trend analysis of major cancer statistics according to sex and severity levels in Korea
Source: PLoS One. 2018 Sep 13;13(9):e0203110. doi: 10.1371/journal.pone.0203110 (PMC6136735; doi:10.1371/journal.pone.0203110)
Supplement: S1 File — (DOCX) [file pone.0203110.s001.docx]

* Number of incident cancer cases in men

| Cancer | Stage | 2006 | | 2007 | | 2008 | | 2009 | | 2010 | | 2011 | | 2012 | | 2013 | |
| --- | --- | --- | --- | --- | --- | --- | --- | --- | --- | --- | --- | --- | --- | --- | --- | --- | --- |
|  |  | N | Rate* | N | Rate* | N | Rate* | N | Rate* | N | Rate* | N | Rate* | N | Rate* | N | Rate* |
| Hepatocellular carcinoma | Local | 4,516 | 31.9 | 4,523 | 31.3 | 5,249 | 35.7 | 5,159 | 34.4 | 5,249 | 34.3 | 5,633 | 34.3 | 5,576 | 35.1 | 5,530 | 34.3 |
|  | Regional | 2,167 | 15.3 | 2,358 | 16.3 | 2,643 | 18.0 | 3,003 | 20.0 | 3,040 | 19.9 | 3,092 | 19.9 | 3,018 | 19.0 | 3,040 | 18.8 |
|  | Distant | 1,319 | 9.3 | 1,475 | 10.2 | 1,621 | 11.0 | 1,736 | 11.6 | 1,896 | 12.4 | 1,899 | 12.4 | 1,914 | 12.1 | 1,820 | 11.3 |
|  | Unknown | 3,234 | 22.8 | 3,193 | 22.1 | 2,439 | 16.6 | 2,151 | 14.3 | 1,909 | 12.5 | 1,712 | 12.5 | 1,715 | 10.8 | 1,715 | 10.6 |
|  | Total | 11,236 | 79.4 | 11,549 | 80.0 | 11,952 | 81.3 | 12,049 | 80.4 | 12,094 | 79.1 | 12,336 | 79.1 | 12,223 | 77.0 | 12,105 | 75.1 |
| Thyroid cancer | Local | 843 | 6.0 | 1,172 | 8.1 | 1,577 | 10.7 | 1,887 | 12.6 | 2,316 | 15.2 | 2,668 | 15.2 | 3,008 | 19.0 | 3,011 | 18.7 |
|  | Regional | 1,058 | 7.5 | 1,532 | 10.6 | 2,187 | 14.9 | 2,604 | 17.4 | 3,424 | 22.4 | 3,897 | 22.4 | 4,553 | 28.7 | 4,841 | 30.0 |
|  | Distant | 61 | 0.4 | 67 | 0.5 | 72 | 0.5 | 82 | 0.5 | 97 | 0.6 | 85 | 0.6 | 79 | 0.5 | 69 | 0.4 |
|  | Unknown | 367 | 2.6 | 412 | 2.9 | 497 | 3.4 | 661 | 4.4 | 504 | 3.3 | 451 | 3.3 | 493 | 3.1 | 533 | 3.3 |
|  | Total | 2,329 | 16.4 | 3,183 | 22.1 | 4,333 | 29.5 | 5,234 | 34.9 | 6,341 | 41.5 | 7,101 | 41.5 | 8,133 | 51.3 | 8,454 | 52.4 |
| Colorectal cancer | Local | 3,740 | 26.4 | 4,279 | 29.7 | 4,885 | 33.2 | 5,756 | 38.4 | 6,272 | 41.0 | 6,888 | 41.0 | 7,327 | 46.2 | 6,411 | 39.7 |
|  | Regional | 4,345 | 30.7 | 4,827 | 33.4 | 5,324 | 36.2 | 5,899 | 39.4 | 6,361 | 41.6 | 6,725 | 41.6 | 6,766 | 42.6 | 6,884 | 42.7 |
|  | Distant | 1,640 | 11.6 | 1,730 | 12.0 | 2,065 | 14.0 | 2,245 | 15.0 | 2,279 | 14.9 | 2,473 | 14.9 | 2,441 | 15.4 | 2,354 | 14.6 |
|  | Unknown | 1,929 | 13.6 | 1,917 | 13.3 | 1,602 | 10.9 | 1,453 | 9.7 | 1,182 | 7.7 | 1,290 | 7.7 | 1,063 | 6.7 | 944 | 5.9 |
|  | Total | 11,654 | 82.3 | 12,753 | 88.4 | 13,876 | 94.4 | 15,353 | 102.4 | 16,094 | 105.3 | 17,376 | 105.3 | 17,597 | 110.9 | 16,593 | 102.9 |
| Gastric cancer | Local | 7,951 | 56.2 | 8,190 | 56.8 | 9,757 | 66.4 | 10,980 | 73.2 | 11,589 | 75.8 | 13,030 | 75.8 | 12,796 | 80.6 | 12,625 | 78.3 |
|  | Regional | 4,650 | 32.8 | 4,687 | 32.5 | 4,814 | 32.7 | 4,848 | 32.3 | 5,163 | 33.8 | 4,826 | 33.8 | 4,610 | 29.1 | 4,447 | 27.6 |
|  | Distant | 2,139 | 15.1 | 2,223 | 15.4 | 2,407 | 16.4 | 2,389 | 15.9 | 2,386 | 15.6 | 2,364 | 15.6 | 2,320 | 14.6 | 2,104 | 13.0 |
|  | Unknown | 2,998 | 21.2 | 2,777 | 19.2 | 2,122 | 14.4 | 1,897 | 12.7 | 1,382 | 9.0 | 1,285 | 9.0 | 1,207 | 7.6 | 1,090 | 6.8 |
|  | Total | 17,738 | 125.3 | 17,877 | 123.9 | 19,100 | 129.9 | 20,114 | 134.2 | 20,520 | 134.3 | 21,505 | 134.3 | 20,933 | 131.9 | 20,266 | 125.7 |
| Lung cancer | Local | 2,190 | 15.5 | 2,313 | 16.0 | 2,379 | 16.2 | 2,517 | 16.8 | 2,670 | 17.5 | 2,878 | 17.5 | 3,066 | 19.3 | 3,000 | 18.6 |
|  | Regional | 3,121 | 22.0 | 3,199 | 22.2 | 3,615 | 24.6 | 3,925 | 26.2 | 4,283 | 28.0 | 4,365 | 28.0 | 4,327 | 27.3 | 4,386 | 27.2 |
|  | Distant | 4,280 | 30.2 | 4,554 | 31.6 | 5,234 | 35.6 | 5,624 | 37.5 | 6,197 | 40.5 | 6,361 | 40.5 | 6,417 | 40.4 | 7,065 | 43.8 |
|  | Unknown | 3,089 | 21.8 | 3,128 | 21.7 | 2,382 | 16.2 | 2,180 | 14.5 | 1,818 | 11.9 | 1,801 | 11.9 | 1,687 | 10.6 | 1,720 | 10.7 |
|  | Total | 12,680 | 89.6 | 13,194 | 91.4 | 13,610 | 92.6 | 14,246 | 95.0 | 14,968 | 97.9 | 15,405 | 97.9 | 15,497 | 97.7 | 16,171 | 100.3 |
| Prostate cancer | Local | 2,135 | 39.6 | 2,777 | 49.0 | 3,534 | 59.3 | 4,107 | 65.5 | 4,694 | 71.1 | 5,240 | 71.1 | 5,279 | 72.5 | 5,229 | 68.9 |
|  | Regional | 640 | 11.9 | 918 | 16.2 | 1,264 | 21.2 | 1,516 | 24.2 | 1,675 | 25.4 | 1,895 | 25.4 | 1,964 | 27.0 | 2,259 | 29.8 |
|  | Distant | 493 | 9.1 | 517 | 9.1 | 597 | 10.0 | 665 | 10.6 | 687 | 10.4 | 797 | 10.4 | 854 | 11.7 | 839 | 11.1 |
|  | Unknown | 1,233 | 22.9 | 1,330 | 23.5 | 1,214 | 20.4 | 1,218 | 19.4 | 1,022 | 15.5 | 1,126 | 15.5 | 1,234 | 16.9 | 1,188 | 15.7 |
|  | Total | 4,501 | 83.5 | 5,542 | 97.8 | 6,609 | 110.9 | 7,506 | 119.7 | 8,078 | 122.3 | 9,058 | 122.3 | 9,331 | 128.1 | 9,515 | 125.4 |
| * Per 100,000 population | | | | | | | | | | | | | | | | | |

* Mean incidence rates and 95% confidence intervals in men

| Cancer | Stage | Mean rate*  (2006-2013) | 95% Confidence interval | | p-trend |
| --- | --- | --- | --- | --- | --- |
|  |  |  | Lower | Upper |  |
| Hepatocellular carcinoma | Local | 34.2 | 30.3 | 38.6 | <0.001 |
|  | Regional | 18.4 | 15.5 | 21.6 | <0.001 |
|  | Distant | 11.3 | 9.0 | 13.8 | <0.001 |
|  | Unknown | 15.1 | 12.6 | 18.1 | <0.001 |
|  | Total | 79.1 | 73.1 | 85.5 | 0.065 |
| Thyroid cancer | Local | 13.5 | 11.1 | 16.3 | 0.834 |
|  | Regional | 19.6 | 16.7 | 22.9 | <0.001 |
|  | Distant | 0.5 | 0.1 | 1.3 | <0.001 |
|  | Unknown | 3.3 | 2.1 | 4.8 | <0.001 |
|  | Total | 36.9 | 32.8 | 41.3 | <0.001 |
| Colorectal cancer | Local | 37.5 | 33.4 | 42.0 | <0.001 |
|  | Regional | 38.9 | 34.7 | 43.4 | <0.001 |
|  | Distant | 14.3 | 11.8 | 17.1 | 0.803 |
|  | Unknown | 9.5 | 7.5 | 11.9 | <0.001 |
|  | Total | 100.0 | 93.2 | 107.2 | 0.007 |
| Gastric cancer | Local | 71.6 | 65.9 | 77.7 | <0.001 |
|  | Regional | 31.5 | 27.7 | 35.6 | <0.001 |
|  | Distant | 15.3 | 12.7 | 18.2 | <0.001 |
|  | Unknown | 12.4 | 10.1 | 15.1 | <0.001 |
|  | Total | 130.8 | 122.9 | 138.9 | 0.343 |
| Lung cancer | Local | 17.3 | 14.6 | 20.5 | <0.001 |
|  | Regional | 25.8 | 22.4 | 29.5 | <0.001 |
|  | Distant | 37.6 | 33.5 | 42.1 | <0.001 |
|  | Unknown | 14.9 | 12.3 | 17.8 | <0.001 |
|  | Total | 95.6 | 89.0 | 102.6 | 0.001 |
| Prostate cancer | Local | 63.2 | 57.7 | 68.9 | <0.001 |
|  | Regional | 23.0 | 19.8 | 26.6 | <0.001 |
|  | Distant | 10.5 | 8.4 | 13.0 | <0.001 |
|  | Unknown | 18.9 | 16.0 | 22.1 | <0.001 |
|  | Total | 115.6 | 108.3 | 123.3 | 0.005 |
| * Per 100,000 population | | | | | |

* Number of incident cancer cases in women

| Cancer | Stage | 2006 | | 2007 | | 2008 | | 2009 | | 2010 | | 2011 | | 2012 | | 2013 | |
| --- | --- | --- | --- | --- | --- | --- | --- | --- | --- | --- | --- | --- | --- | --- | --- | --- | --- |
|  |  | N | Rate* | N | Rate* | N | Rate* | N | Rate* | N | Rate* | N | Rate* | N | Rate* | N | Rate* |
| Hepatocellular carcinoma | Local | 1,489 | 10.0 | 1,527 | 10.1 | 1,664 | 10.8 | 1,739 | 11.1 | 1,804 | 11.3 | 1,929 | 11.8 | 1,783 | 10.7 | 1,779 | 10.5 |
|  | Regional | 657 | 4.4 | 686 | 4.5 | 780 | 5.1 | 851 | 5.4 | 919 | 5.7 | 971 | 5.9 | 913 | 5.5 | 932 | 5.5 |
|  | Distant | 439 | 3.0 | 466 | 3.1 | 562 | 3.6 | 627 | 4.0 | 649 | 4.1 | 688 | 4.2 | 691 | 4.2 | 649 | 3.8 |
|  | Unknown | 1,181 | 7.9 | 1,248 | 8.2 | 939 | 6.1 | 873 | 5.6 | 827 | 5.2 | 760 | 4.7 | 753 | 4.5 | 727 | 4.3 |
|  | Total | 3,766 | 25.3 | 3,927 | 25.9 | 3,945 | 25.6 | 4,090 | 26.0 | 4,199 | 26.2 | 4,348 | 26.6 | 4,140 | 24.9 | 4,087 | 24.2 |
| Thyroid cancer | Local | 6,065 | 40.8 | 7,974 | 52.6 | 10,182 | 66.0 | 11,827 | 75.3 | 13,187 | 82.3 | 15,357 | 94.1 | 16,610 | 100.0 | 14,716 | 87.1 |
|  | Regional | 5,556 | 37.4 | 7,572 | 50.0 | 10,238 | 66.4 | 12,115 | 77.1 | 14,776 | 92.2 | 16,344 | 100.1 | 17,671 | 106.3 | 17,101 | 101.3 |
|  | Distant | 148 | 1.0 | 180 | 1.2 | 195 | 1.3 | 225 | 1.4 | 266 | 1.7 | 182 | 1.1 | 213 | 1.3 | 163 | 1.0 |
|  | Unknown | 2,034 | 13.7 | 2,353 | 15.5 | 2,361 | 15.3 | 3,055 | 19.4 | 2,117 | 13.2 | 2,216 | 13.6 | 1,867 | 11.2 | 2,107 | 12.5 |
|  | Total | 13,803 | 92.8 | 18,079 | 119.3 | 22,976 | 149.0 | 27,222 | 173.3 | 30,346 | 189.4 | 34,099 | 208.9 | 36,361 | 218.8 | 34,087 | 201.9 |
| Colorectal cancer | Local | 2,375 | 16.0 | 2,646 | 17.5 | 2,964 | 19.2 | 3,440 | 21.9 | 3,671 | 22.9 | 3,949 | 24.2 | 4,278 | 25.7 | 3,843 | 22.8 |
|  | Regional | 3,098 | 20.8 | 3,337 | 22.0 | 3,697 | 24.0 | 4,009 | 25.5 | 4,290 | 26.8 | 4,582 | 28.1 | 4,671 | 28.1 | 4,627 | 27.4 |
|  | Distant | 1,225 | 8.2 | 1,329 | 8.8 | 1,499 | 9.7 | 1,590 | 10.1 | 1,631 | 10.2 | 1,654 | 10.1 | 1,809 | 10.9 | 1,744 | 10.3 |
|  | Unknown | 1,526 | 10.3 | 1,440 | 9.5 | 1,136 | 7.4 | 1,064 | 6.8 | 879 | 5.5 | 926 | 5.7 | 895 | 5.4 | 811 | 4.8 |
|  | Total | 8,224 | 55.3 | 8,752 | 57.8 | 9,296 | 60.3 | 10,103 | 64.3 | 10,471 | 65.4 | 11,111 | 68.1 | 11,653 | 70.1 | 11,025 | 65.3 |
| Gastric cancer | Local | 3,760 | 25.3 | 3,978 | 26.3 | 4,568 | 29.6 | 5,327 | 33.9 | 5,558 | 34.7 | 5,982 | 36.6 | 5,938 | 35.7 | 5,827 | 34.5 |
|  | Regional | 2,168 | 14.6 | 2,247 | 14.8 | 2,368 | 15.4 | 2,336 | 14.9 | 2,464 | 15.4 | 2,434 | 14.9 | 2,208 | 13.3 | 2,319 | 13.7 |
|  | Distant | 1,072 | 7.2 | 1,076 | 7.1 | 1,171 | 7.6 | 1,141 | 7.3 | 1,178 | 7.4 | 1,134 | 6.9 | 1,132 | 6.8 | 1,048 | 6.2 |
|  | Unknown | 1,692 | 11.4 | 1,633 | 10.8 | 1,185 | 7.7 | 1,093 | 7.0 | 922 | 5.8 | 840 | 5.1 | 799 | 4.8 | 724 | 4.3 |
|  | Total | 8,692 | 58.4 | 8,934 | 59.0 | 9,292 | 60.2 | 9,897 | 63.0 | 10,122 | 63.2 | 10,390 | 63.7 | 10,077 | 60.6 | 9,918 | 58.7 |
| Lung cancer | Local | 907 | 6.1 | 1,001 | 6.6 | 1,053 | 6.8 | 1,170 | 7.4 | 1,305 | 8.1 | 1,510 | 9.3 | 1,738 | 10.5 | 1,730 | 10.2 |
|  | Regional | 911 | 6.1 | 938 | 6.2 | 1,123 | 7.3 | 1,145 | 7.3 | 1,309 | 8.2 | 1,443 | 8.8 | 1,427 | 8.6 | 1,465 | 8.7 |
|  | Distant | 1,822 | 12.2 | 1,903 | 12.6 | 2,226 | 14.4 | 2,388 | 15.2 | 2,631 | 16.4 | 2,765 | 16.9 | 2,769 | 16.7 | 2,921 | 17.3 |
|  | Unknown | 1,405 | 9.4 | 1,400 | 9.2 | 1,130 | 7.3 | 1,096 | 7.0 | 1,013 | 6.3 | 995 | 6.1 | 904 | 5.4 | 890 | 5.3 |
|  | Total | 5,045 | 33.9 | 5,242 | 34.6 | 5,532 | 35.9 | 5,799 | 36.9 | 6,258 | 39.1 | 6,713 | 41.1 | 6,838 | 41.1 | 7,006 | 41.5 |
| Breast cancer | Local | 4,968 | 33.4 | 5,734 | 37.8 | 6,416 | 41.6 | 7,334 | 46.7 | 8,062 | 50.3 | 9,220 | 56.5 | 9,623 | 57.9 | 9,989 | 59.2 |
|  | Regional | 3,433 | 23.1 | 3,846 | 25.4 | 4,306 | 27.9 | 4,704 | 29.9 | 5,213 | 32.5 | 5,448 | 33.4 | 5,593 | 33.7 | 5,888 | 34.9 |
|  | Distant | 446 | 3.0 | 524 | 3.5 | 608 | 3.9 | 752 | 4.8 | 679 | 4.2 | 758 | 4.6 | 810 | 4.9 | 748 | 4.4 |
|  | Unknown | 2,033 | 13.7 | 1,860 | 12.3 | 1,431 | 9.3 | 796 | 5.1 | 615 | 3.8 | 628 | 3.8 | 589 | 3.5 | 606 | 3.6 |
|  | Total | 10,880 | 73.1 | 11,964 | 79.0 | 12,761 | 82.7 | 13,586 | 86.5 | 14,569 | 90.9 | 16,054 | 98.4 | 16,615 | 100.0 | 17,231 | 102.0 |
| Cervical cancer | Local | 2,131 | 14.3 | 2,039 | 13.5 | 2,165 | 14.0 | 2,117 | 13.5 | 2,309 | 14.4 | 2,202 | 13.5 | 2,083 | 12.5 | 1,981 | 11.7 |
|  | Regional | 852 | 5.7 | 907 | 6.0 | 1,042 | 6.8 | 1,018 | 6.5 | 1,049 | 6.5 | 966 | 5.9 | 987 | 5.9 | 1,092 | 6.5 |
|  | Distant | 197 | 1.3 | 237 | 1.6 | 260 | 1.7 | 254 | 1.6 | 300 | 1.9 | 301 | 1.8 | 293 | 1.8 | 318 | 1.9 |
|  | Unknown | 872 | 5.9 | 579 | 3.8 | 541 | 3.5 | 417 | 2.7 | 307 | 1.9 | 302 | 1.9 | 256 | 1.5 | 242 | 1.4 |
|  | Total | 4,052 | 27.2 | 3,762 | 24.8 | 4,008 | 26.0 | 3,806 | 24.2 | 3,965 | 24.8 | 3,771 | 23.1 | 3,619 | 21.8 | 3,633 | 21.5 |
| * Per 100,000 population | | | | | | | | | | | | | | | | | |

* Mean incidence rates and 95% confidence intervals in women

| Cancer | Stage | Mean rate (2006-2013) | 95% Confidence interval | | p-trend |
| --- | --- | --- | --- | --- | --- |
|  |  |  | Lower | Upper |  |
| Hepatocellular carcinoma | Local | 10.6 | 8.5 | 13.1 | <0.001 |
|  | Regional | 5.3 | 3.8 | 7.1 | <0.001 |
|  | Distant | 3.8 | 2.5 | 5.4 | <0.001 |
|  | Unknown | 5.8 | 4.2 | 7.7 | <0.001 |
|  | Total | 25.4 | 22.0 | 29.1 | 0.366 |
| Thyroid cancer | Local | 74.8 | 68.9 | 81.0 | 0.184 |
|  | Regional | 78.9 | 72.8 | 85.3 | <0.001 |
|  | Distant | 1.3 | 0.6 | 2.3 | <0.001 |
|  | Unknown | 14.3 | 11.8 | 17.1 | <0.001 |
|  | Total | 169.1 | 160.2 | 178.4 | 0.001 |
| Colorectal cancer | Local | 21.3 | 18.2 | 24.7 | <0.001 |
|  | Regional | 25.4 | 22.0 | 29.1 | <0.001 |
|  | Distant | 9.8 | 7.7 | 12.2 | 0.438 |
|  | Unknown | 6.9 | 5.2 | 8.9 | <0.001 |
|  | Total | 63.4 | 58.0 | 69.1 | 0.003 |
| Gastric cancer | Local | 32.1 | 28.3 | 36.3 | <0.001 |
|  | Regional | 14.6 | 12.1 | 17.5 | <0.001 |
|  | Distant | 7.1 | 5.4 | 9.2 | <0.001 |
|  | Unknown | 7.1 | 5.4 | 9.2 | <0.001 |
|  | Total | 60.9 | 55.6 | 66.5 | 0.499 |
| Lung cancer | Local | 8.1 | 6.3 | 10.4 | <0.001 |
|  | Regional | 7.6 | 5.8 | 9.8 | <0.001 |
|  | Distant | 15.3 | 12.7 | 18.2 | <0.001 |
|  | Unknown | 7.0 | 5.3 | 9.1 | <0.001 |
|  | Total | 38.0 | 33.8 | 42.5 | <0.001 |
| Breast cancer | Local | 47.9 | 43.2 | 52.9 | <0.001 |
|  | Regional | 30.1 | 26.4 | 34.2 | <0.001 |
|  | Distant | 4.1 | 2.8 | 5.8 | 0.457 |
|  | Unknown | 6.9 | 5.2 | 8.9 | <0.001 |
|  | Total | 89.1 | 82.7 | 95.9 | <0.001 |
| Cervical cancer | Local | 13.4 | 11.0 | 16.2 | <0.001 |
|  | Regional | 6.3 | 4.6 | 8.2 | <0.001 |
|  | Distant | 1.8 | 1.0 | 2.9 | <0.001 |
|  | Unknown | 2.9 | 1.8 | 4.3 | <0.001 |
|  | Total | 24.1 | 20.8 | 27.8 | 0.001 |
| * Per 100,000 population | | | | | |
