# Supplementary material for: Trend analysis of major cancer statistics according to sex and severity levels in Korea
Source: PLoS One. 2018 Sep 13;13(9):e0203110. doi: 10.1371/journal.pone.0203110 (PMC6136735; doi:10.1371/journal.pone.0203110)
Supplement: S2 File — (DOCX) [file pone.0203110.s002.docx]

* Absolute survival rates of up to eight years in men

|  |  | 0Y | 1Y | 2Y | 3Y | 4Y | 5Y | 6Y | 7Y | 8Y |
| --- | --- | --- | --- | --- | --- | --- | --- | --- | --- | --- |
| Localized | Hepatocellular carcinoma | 100% | 66.8% | 52.3% | 43.3% | 36.5% | 32.2% | 28.4% | 25.9% | 23.6% |
|  | Thyroid cancer | 100% | 98.8% | 97.4% | 96.8% | 96.3% | 95.3% | 94.5% | 94.0% | 93.8% |
|  | Colorectal cancer | 100% | 94.0% | 90.3% | 86.7% | 84.1% | 81.5% | 79.2% | 77.6% | 75.5% |
|  | Gastric cancer | 100% | 92.6% | 89.0% | 86.0% | 83.3% | 81.1% | 78.6% | 76.4% | 74.2% |
|  | Lung cancer | 100% | 62.3% | 46.5% | 38.5% | 34.2% | 31.9% | 29.7% | 27.8% | 26.1% |
|  | Prostate cancer | 100% | 96.0% | 91.3% | 87.2% | 82.6% | 78.2% | 74.0% | 69.8% | 66.3% |
|  |  | 0Y | 1Y | 2Y | 3Y | 4Y | 5Y | 6Y | 7Y | 8Y |
| Regional | Hepatocellular carcinoma | 100% | 38.4% | 24.3% | 17.8% | 14.1% | 11.3% | 9.6% | 8.3% | 7.4% |
|  | Thyroid cancer | 100% | 98.0% | 97.3% | 96.4% | 95.6% | 95.2% | 94.9% | 93.3% | 92.1% |
|  | Colorectal cancer | 100% | 92.1% | 84.2% | 77.8% | 72.8% | 68.5% | 65.3% | 62.9% | 60.4% |
|  | Gastric cancer | 100% | 79.1% | 64.8% | 56.5% | 51.1% | 47.7% | 45.2% | 43.1% | 41.4% |
|  | Lung cancer | 100% | 55.7% | 34.8% | 26.2% | 22.6% | 20.0% | 18.6% | 16.9% | 15.4% |
|  | Prostate cancer | 100% | 94.8% | 88.1% | 81.1% | 76.8% | 72.0% | 67.6% | 64.1% | 61.4% |
|  |  | 0Y | 1Y | 2Y | 3Y | 4Y | 5Y | 6Y | 7Y | 8Y |
| Distant | Hepatocellular carcinoma | 100% | 20.0% | 10.6% | 6.8% | 4.8% | 3.2% | 2.7% | 2.0% | 1.8% |
|  | Thyroid cancer | 100% | 69.1% | 65.5% | 58.2% | 56.4% | 56.4% | 54.5% | 49.1% | 45.5% |
|  | Colorectal cancer | 100% | 62.1% | 39.6% | 26.9% | 21.0% | 17.2% | 15.2% | 13.8% | 12.6% |
|  | Gastric cancer | 100% | 36.4% | 17.2% | 10.7% | 8.1% | 6.5% | 6.0% | 5.6% | 5.1% |
|  | Lung cancer | 100% | 21.6% | 10.9% | 6.6% | 5.0% | 4.4% | 3.8% | 3.3% | 3.1% |
|  | Prostate cancer | 100% | 76.7% | 58.0% | 43.2% | 34.9% | 27.9% | 24.3% | 20.2% | 16.8% |
|  |  | 0Y | 1Y | 2Y | 3Y | 4Y | 5Y | 6Y | 7Y | 8Y |
| Unknown | Hepatocellular carcinoma | 100% | 54.4% | 43.1% | 36.5% | 32.6% | 29.5% | 27.1% | 25.3% | 24.0% |
|  | Thyroid cancer | 100% | 96.0% | 94.6% | 92.9% | 92.0% | 90.9% | 89.5% | 88.6% | 87.5% |
|  | Colorectal cancer | 100% | 80.9% | 71.6% | 66.3% | 62.1% | 59.1% | 55.9% | 53.7% | 51.6% |
|  | Gastric cancer | 100% | 70.7% | 61.3% | 55.4% | 52.2% | 50.0% | 48.0% | 46.4% | 44.3% |
|  | Lung cancer | 100% | 48.7% | 33.4% | 28.3% | 25.8% | 24.3% | 23.2% | 22.3% | 21.4% |
|  | Prostate cancer | 100% | 93.2% | 85.6% | 79.1% | 74.1% | 69.4% | 64.6% | 60.3% | 54.9% |

* Absolute survival rates of up to eight years in women

|  |  | 0Y | 1Y | 2Y | 3Y | 4Y | 5Y | 6Y | 7Y | 8Y |
| --- | --- | --- | --- | --- | --- | --- | --- | --- | --- | --- |
| Localized | Hepatocellular carcinoma | 100% | 67.6% | 54.8% | 45.5% | 39.0% | 34.8% | 32.3% | 29.8% | 27.4% |
|  | Thyroid cancer | 100% | 99.7% | 99.6% | 99.3% | 99.0% | 98.7% | 98.5% | 98.1% | 97.6% |
|  | Colorectal cancer | 100% | 94.4% | 90.8% | 88.0% | 85.7% | 83.9% | 81.9% | 80.3% | 78.4% |
|  | Gastric cancer | 100% | 92.5% | 89.5% | 86.9% | 84.9% | 83.5% | 81.7% | 80.4% | 79.1% |
|  | Lung cancer | 100% | 75.1% | 63.1% | 56.1% | 51.7% | 48.1% | 45.8% | 44.2% | 42.8% |
|  | Breast cancer | 100% | 99.3% | 98.5% | 97.5% | 96.4% | 95.7% | 94.9% | 94.0% | 93.2% |
|  | Cervical cancer | 100% | 96.8% | 94.0% | 91.9% | 90.3% | 88.7% | 87.5% | 85.8% | 84.7% |
|  |  | 0Y | 1Y | 2Y | 3Y | 4Y | 5Y | 6Y | 7Y | 8Y |
| Regional | Hepatocellular carcinoma | 100% | 37.9% | 26.9% | 18.8% | 14.1% | 12.2% | 10.6% | 9.8% | 8.9% |
|  | Thyroid cancer | 100% | 99.4% | 99.1% | 98.9% | 98.5% | 98.1% | 97.8% | 97.5% | 97.1% |
|  | Colorectal cancer | 100% | 92.1% | 84.5% | 78.8% | 73.9% | 71.0% | 68.7% | 66.7% | 64.7% |
|  | Gastric cancer | 100% | 77.9% | 65.8% | 58.6% | 54.5% | 51.4% | 49.2% | 46.8% | 45.8% |
|  | Lung cancer | 100% | 64.0% | 48.4% | 38.1% | 34.1% | 31.3% | 27.2% | 24.9% | 22.6% |
|  | Breast cancer | 100% | 98.3% | 95.7% | 92.5% | 89.4% | 87.1% | 85.3% | 83.3% | 81.8% |
|  | Cervical cancer | 100% | 90.7% | 81.5% | 75.2% | 71.5% | 67.5% | 64.8% | 63.5% | 61.9% |
|  |  | 0Y | 1Y | 2Y | 3Y | 4Y | 5Y | 6Y | 7Y | 8Y |
| Distant | Hepatocellular carcinoma | 100% | 18.8% | 9.0% | 4.4% | 2.5% | 2.3% | 2.3% | 2.3% | 1.9% |
|  | Thyroid cancer | 100% | 81.8% | 79.6% | 77.4% | 75.2% | 70.8% | 67.9% | 65.7% | 65.0% |
|  | Colorectal cancer | 100% | 60.1% | 37.6% | 27.2% | 21.0% | 18.5% | 16.6% | 14.8% | 14.0% |
|  | Gastric cancer | 100% | 35.6% | 15.8% | 9.9% | 7.5% | 6.2% | 5.5% | 4.7% | 4.6% |
|  | Lung cancer | 100% | 41.9% | 25.1% | 16.1% | 10.4% | 7.2% | 5.5% | 4.6% | 3.4% |
|  | Breast cancer | 100% | 84.1% | 70.8% | 55.6% | 43.5% | 36.0% | 29.8% | 24.8% | 21.4% |
|  | Cervical cancer | 100% | 62.0% | 39.1% | 32.8% | 28.6% | 27.1% | 23.4% | 19.3% | 16.7% |
|  |  | 0Y | 1Y | 2Y | 3Y | 4Y | 5Y | 6Y | 7Y | 8Y |
| Unknown | Hepatocellular carcinoma | 100% | 53.0% | 43.5% | 37.8% | 33.7% | 30.4% | 28.2% | 26.4% | 24.5% |
|  | Thyroid cancer | 100% | 98.0% | 97.2% | 96.5% | 95.9% | 95.5% | 95.0% | 94.5% | 94.2% |
|  | Colorectal cancer | 100% | 78.6% | 70.9% | 64.9% | 60.7% | 58.1% | 56.9% | 55.8% | 54.9% |
|  | Gastric cancer | 100% | 70.5% | 59.9% | 54.1% | 50.8% | 49.0% | 47.4% | 46.0% | 44.7% |
|  | Lung cancer | 100% | 59.9% | 48.0% | 42.8% | 39.3% | 37.5% | 36.4% | 35.1% | 34.5% |
|  | Breast cancer | 100% | 96.6% | 93.2% | 90.7% | 88.8% | 87.5% | 86.2% | 85.2% | 84.0% |
|  | Cervical cancer | 100% | 88.7% | 81.4% | 77.1% | 74.9% | 73.1% | 71.4% | 69.8% | 67.9% |
